# Tumour-associated macrophages exhibit anti-tumoural properties in Sonic Hedgehog medulloblastoma

**DOI:** 10.1038/s41467-019-10458-9

**Published:** 2019-06-03

**Authors:** Victor Maximov, Zhihong Chen, Yun Wei, M. Hope Robinson, Cameron J. Herting, Nithya S. Shanmugam, Vasilisa A. Rudneva, Kelly C. Goldsmith, Tobey J. MacDonald, Paul A. Northcott, Dolores Hambardzumyan, Anna M. Kenney

**Affiliations:** 1 Department of Pediatrics, Neuro-Oncology Division and Aflac Cancer and Blood Disorders Center of Children’s Healthcare of Atlanta, Emory University, Atlanta, 30322 GA United States; 2 Winship Cancer Institute, Emory University, Atlanta, 30322 GA USA; 3 Graduate Division of Molecular and Systems Pharmacology, Emory University, Atlanta, 30322 GA USA; 4 St. Jude Children’s Research Hospital, Memphis, 38105 TN USA

**Keywords:** CNS cancer, Paediatric cancer, Tumour immunology

## Abstract

Medulloblastoma, which is the most common malignant paediatric brain tumour, has a 70% survival rate, but standard treatments often lead to devastating life-long side effects and recurrence is fatal. One of the emerging strategies in the search for treatments is to determine the roles of tumour microenvironment cells in the growth and maintenance of tumours. The most attractive target is tumour-associated macrophages (TAMs), which are abundantly present in the Sonic Hedgehog (SHH) subgroup of medulloblastoma. Here, we report an unexpected beneficial role of TAMs in SHH medulloblastoma. In human patients, decreased macrophage number is correlated with significantly poorer outcome. We confirm macrophage anti-tumoural behaviour in both ex vivo and in vivo murine models of SHH medulloblastoma. Taken together, our findings suggest that macrophages play a positive role by impairing tumour growth in medulloblastoma, in contrast to the pro-tumoural role played by TAMs in glioblastoma, another common brain tumour.

## Abstract (web summary)

The Sonic Hedgehog subgroup of medulloblastoma are characterised by the high infiltration of tumour-associated macrophages (TAMs). Here, the authors show that TAM numbers in patients are associated with better prognosis and that, consistently, in a murine model of medulloblastoma, these TAMs have anti-tumoural properties.

## Introduction

Grade IV medulloblastoma is the most common solid malignant tumour of children and is a leading cause of paediatric mortality^1,2^. Current treatment includes surgery, chemotherapy and cranio-spinal irradiation, resulting in a 5-year overall survival rate of 70%; however, survivors are frequently left with life-long sequelae, including cognitive impairment, seizures, premature aging and increased susceptibility to other cancers. The recent classification of medulloblastoma by molecular and genetic profiling resulted in identification of several distinct subgroups^1^. The most prevalent subclass of medulloblastoma is Sonic Hedgehog (SHH), comprising 30% of all cases. SHH medulloblastoma is thought to arise upon mutation in cerebellar granular neuronal precursors, whose developmental expansion requires signalling by SHH, which is a secreted ligand produced by the neighbouring Purkinje neurons^3^. The disease has been modelled in mice, with tumours that closely recapitulate human disease, providing a relevant system for studying medulloblastoma in vivo^4,5^.

In recent years, the role played by the tumour microenvironment (TME) in promoting or impairing tumour growth has garnered significant attention. Tumour-associated macrophages (TAMs) are a key component of the TME, and they can contribute to tumour immune system evasion, suppress T-cell activity, and support tumour growth by promoting angiogenesis or suppress tumour growth if they are pro-inflammatory^6,7^. Whether TAMs promote or impede tumour growth is tissue type-dependent and still remains controversial. TAMs were shown to be anti-tumoural in stomach, colorectal and melanoma tumours, whereas pro-tumoural effects have been identified in breast, prostate, kidney and other types of cancers^8^. In both human and murine adult glioblastoma, it was shown that TAMs include both blood-circulating monocytes and brain-resident microglia^9^. Recently, it was reported that of all the medulloblastoma subgroups, human SHH has the greatest number of TAMs, as well as increased expression of some macrophage-associated genes^10–12^. However, the role played by TAMs in SHH medulloblastoma is not yet established. There is currently no evidence to support their role, good or bad, in these tumours.

To investigate the role of macrophages in SHH medulloblastoma, we employed a variety of mouse models of *NeuroD2:SmoA1*^5^ as well as pharmaceutical approaches. We show that bone marrow-derived macrophages (BMDMs) delay growth of tumour cells and promote apoptosis in vitro, and we provide evidence that medulloblastoma-associated TAMs exhibit tumour growth-inhibiting properties in vivo. We also developed an ex vivo assay to assess the effects of microglia or BMDMs effect on organotypic tumour slices, which allows us to closely model in vivo effects. Finally, we demonstrate that deletion of the C−C chemokine receptor type-2 gene (*Ccr2*) from the host of allografted tumours, or using a pharmacological approach by treating tumour-bearing mice with two CSF1R inhibitors, significantly decreases survival time of tumour-bearing mice compared to vehicle treatment. Overall, these findings demonstrate that TAMs have an important role in inhibiting tumour growth in medulloblastoma. Further investigation of immune system interactions with tumour cells in medulloblastoma is warranted, as it could provide an intriguing route to the development of novel treatment approaches by leveraging the host immune system.

## Results

### TAM-associated genes are upregulated in human SHH MB

**Fig. 1:**
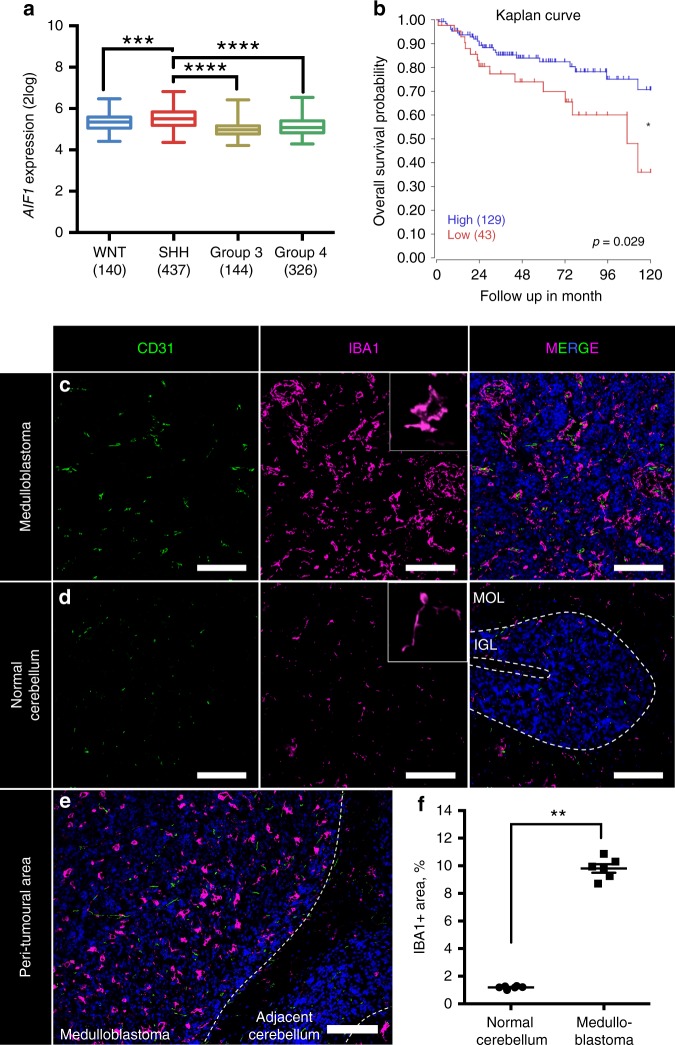
SHH medulloblastoma has pronounced activation of TAMs. **a** Microarray data analysis of human patient cohort for *AIF1* gene (from Cavalli et al.^13^). WNT Wingless (*N* = 140), SHH Sonic Hedgehog (*N* = 437), *N* = 144 for group 3, and *N* = 326 for group 4. Box plots represent median and quratiles with whiskers indicating range of values. One-way ANOVA with multiple comparisons, *F* = 69.25, ***P < 0.001, ****P < 0.0001. **b** Kaplan–Meier graphs of patients with high (*N* = 129, blue) and low (first quartile, *N* = 43, red) *AIF1* expression, Log-rank Mantel–Cox test, *P < 0.05. **c**–**e** Immunofluorescent staining with macrophage marker IBA1 (magenta), endothelial marker CD31 (green), and nuclear marker DAPI (blue) of peri-tumoural area. Scale bars represent 100 µm. **f** Semi-quantitative analysis of macrophage linear density by measuring area of IBA1 staining in normal cerebellum and medulloblastoma; each point represents analysis of at least five images (×20 magnification) from individual animals, mean ± S.E.M., *N* = 6, Mann–Whitney *U* test, **P < 0.01

A previous study showed upregulation of macrophage markers in SHH medulloblastoma^11^. To confirm and elaborate on these findings, we analysed microarray data from a recently published study that analysed tumours of a large cohort of paediatric patients^13^. We queried several TAM-associated genes and compared their expression across the medulloblastoma subtypes. *AIF1* encodes the protein ionised calcium-binding adaptor molecule 1 (IBA1), which is widely used as a cellular marker for macrophages. We found that there was a significant increase in *AIF1* transcription in SHH medulloblastoma, moreso than in any other medulloblastoma subgroup (*P* < 0.0001, one-way ANOVA; Fig. 1a). Similarly, the expression of macrophage markers CD11b and CD68 was also increased in the SHH subgroup (*P* < 0.0001, one-way ANOVA; Supplementary Fig. 2). When we compared survival of SHH medulloblastoma patients, we found significantly worse outcome for patients that had lower expression of the *AIF1* gene (Fig. 1b)^13^. According to the recent re-classification performed by Cavalli et al.^13^, our analysis showed that for the four identified SHH subgroups, only SHHα demonstrated a significant difference in survival between *AIF1* high-expressers and low-expressers (*P* < 0.05, Log-rank Mantel–Cox test; Supplementary Fig. 3). These data suggest that the role of macrophages in SHH MB is protective and that the presence of macrophages can be beneficial for survival.

### Murine MB features pronounced TAM activation

To investigate whether TAMs and their associated genes are similarly increased in the murine model of SHH medulloblastoma, we stained *NeuroD2:SmoA1* tumours with the pan-macrophage marker IBA1. We found that the density of TAMs was significantly increased in the tumours (Fig. 1c) compared to naive cerebellum (Fig. 1d), where microglia can be found maintaining their ramified resting phenotype (small cell bodies and long processes). In the peri-tumoural region (Fig. 1e), however, microglia altered their morphology from ramified to reactive amoeboid phenotype, suggesting transformation to TAMs. We quantified the percentage area of a microscopic field occupied by IBA1^+^ cells and found that they occupied a considerably higher area in the tumour (9.8 ± 0.3%, mean ± S.E.M., *N* = 6) than ramified microglia in naive cerebellum (1.2 ± 0.04%, *N* = 6, Mann–Whitney *U* test, *P* < 0.01) (Fig. 1f). Overall, our results showed that similar to human medulloblastoma, TAMs are activated in the *NeuroD2:SmoA1* murine SHH model, rendering the model suitable to determine the functional role of TAMs in medulloblastoma.

### TAMs are both microglia and myeloid cells in medulloblastoma

**Fig. 2:**
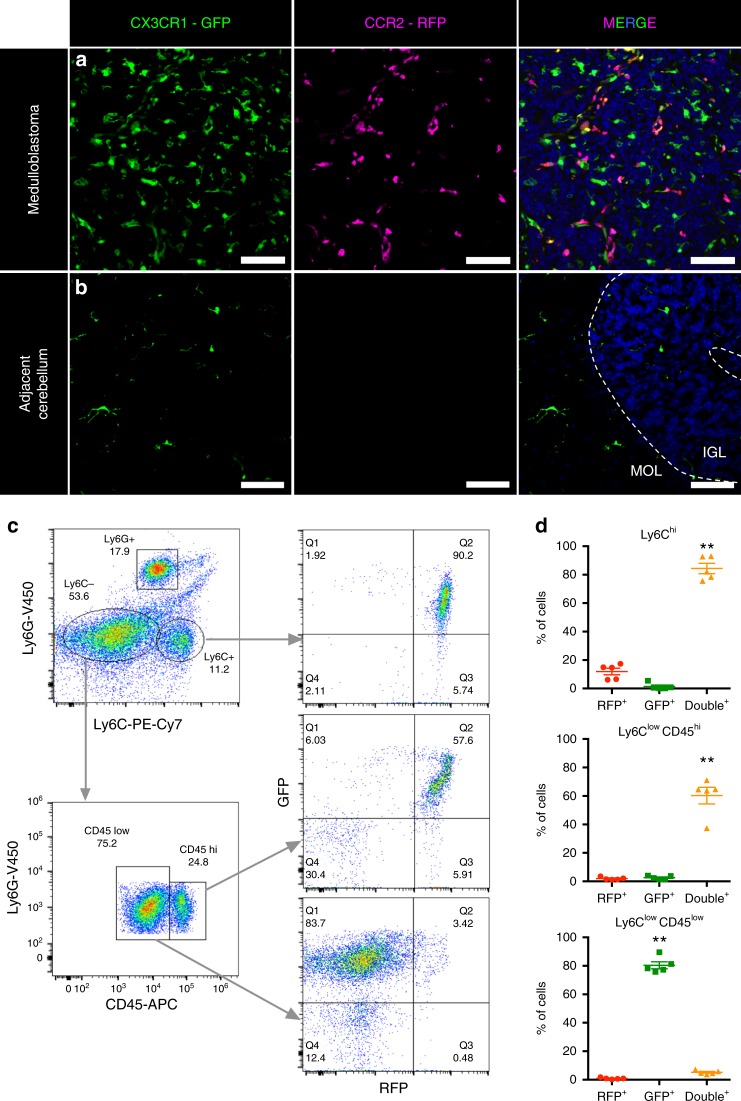
TAMs in medulloblastoma are predominantly of myeloid origin. **a**, **b** Direct fluorescent visualisation of *SmoA1* orthotopic allograft in *Cx3cr1*^*+/GFP*^;*Ccr2*^*+/RFP*^ mouse model revealed the presence of leukocytes in tumour area (**a**), but not in adjacent cerebellum (**b**). Green – GFP, Magenta – RFP, Blue – DAPI. Scale bars represent 100 µm; data are representative of at least five independent experiments. **c** Flow cytometry gating strategy for analysis of immune-cell types in tumours from *Cx3cr1*^*+/GFP*^;*Ccr2*^*+/RFP*^ mice. Ly6C – monocyte marker, Ly6G – neutrophil marker, CD45 – myeloid immune-cell marker. **d** Quantification of data plotted in **c**, mean ± S.E.M., *N* = 5, one-way ANOVA with multiple comparisons, **P < 0.01

Currently, information regarding both immune-cell composition of TME in medulloblastoma and the cellular identity of TAMs in medulloblastoma is limited. To define the cellular identity of TAMs in medulloblastoma, we utilised orthotopic intra-cranial allografts of *SmoA1* medulloblastoma into *Ccr2*^*+/RFP*^*Cx3cr1*^*+/GFP*^ double knock-in mice. In these mice, GFP and RFP replace one copy of each of the *Cx3Cr1* and *Ccr2* genes, respectively^14^. The *Ccr2*^*+/RFP*^*Cx3cr1*^*+/GFP*^ mouse is a valuable tool for studying brain tumours since it allows for distinguishing bone marrow (BM)-derived myeloid cells from brain-resident microglia^15^. Specifically, CX3CR1 is a fractalkine receptor that is produced by microglia, monocytes, and macrophages, whereas CCR2 is exclusively produced by BM-derived myeloid cells. Immunofluorescent analysis revealed the presence of both GFP^+^ and RFP^+^ cells in the allograft tumour, indicating the presence of CCR2^+^ cells in the tumours (Fig. 2a). GFP^+^ cells are microglial cells, and cells that are either RFP^+^ or GFP^+^RFP^+^ indicate infiltrating monocytes (Fig. 2a, right panel). Adjacent control cerebellum only contained GFP^+^ cells, which are naive microglia (Fig. 2b).

To confirm these findings, we performed flow cytometry analysis of these tumours and investigated their immune-cell composition. First, we selected the CD11b^+^CD45^+^ population of immune cells, plotted them by Lymphocyte antigen 6 complex locus C1 (Ly6C) and Lymphocyte antigen 6 complex locus G6D (Ly6G) to distinguish monocytes (Ly6C^+^) and neutrophils (Ly6C^+^Ly6G^+^), followed by further analysis of their RFP and GFP expressions (Fig. 2c, d). We found that Ly6C^hi^ and Ly6C^low^CD45^hi^ subpopulations (newly infiltrated macrophages and TAMs) were GFP^+^RFP^+^ (84 ± 3% and 60 ± 5%, respectively, mean ± S.E.M., *N* = 5), with 12 ± 2% and 2 ± 0.8% of GFP^−^RFP^+^ cells, respectively. Ly6G^+^ cells were GFP^−^RFP^−^ neutrophils. The majority of Ly6C^low^CD45^low^ cells (80 ± 2%) were GFP^+^RFP^−^, confirming properties of microglial cells. Quantification of relative cell composition in *Ccr2*^*+/RFP*^*Cx3cr1*^*+/GFP*^ revealed that microglial cells (35 ± 5%, mean ± S.E.M., *N* = 5) comprised a similar population size compared to infiltrating myeloid cells (37 ± 4%) (Supplementary Fig. 4a).

To define the immune-cell composition of medulloblastoma TME in the control C57BL/6J mice, we performed multi-colour flow cytometry analysis of secondary *SmoA1* tumours. We found that the majority of the cell population in mouse SHH medulloblastoma tumours are tumour cells, whereas TME cells only represent a small population (<2%) of total cells, and about 75% of these TME cells are CD45^+^ cells. Further characterisation of CD45^+^ cells revealed that the most abundant population is infiltrating myeloid cells (72 ± 4%, mean ± S.E.M., *N* = 14), i.e., monocytes and differentiated TAMs, whereas microglia represent 19 ± 3% of total immune-cell population (Supplementary Fig. 4b). In addition, to underscore the unique composition of the immune TME profile of SHH tumours, we compared them to group 3 medulloblastoma^16^. Both subtypes were generated in B6 mice and we found critical differences in the immune-cell populations. Although the majority of immune cells in both tumours were of myeloid origin, medulloblastoma group 3 tumours had a third of infiltrating T-lymphocytes and almost no microglial cells present (Supplementary Fig. 4c). Overall, these experiments demonstrate the infiltration of CD11b^+^CD45^hi^CCR2^+^ inflammatory monocytes in medulloblastoma tumours.

### CCL2 is elevated in murine and human medulloblastoma tumours

**Fig. 3:**
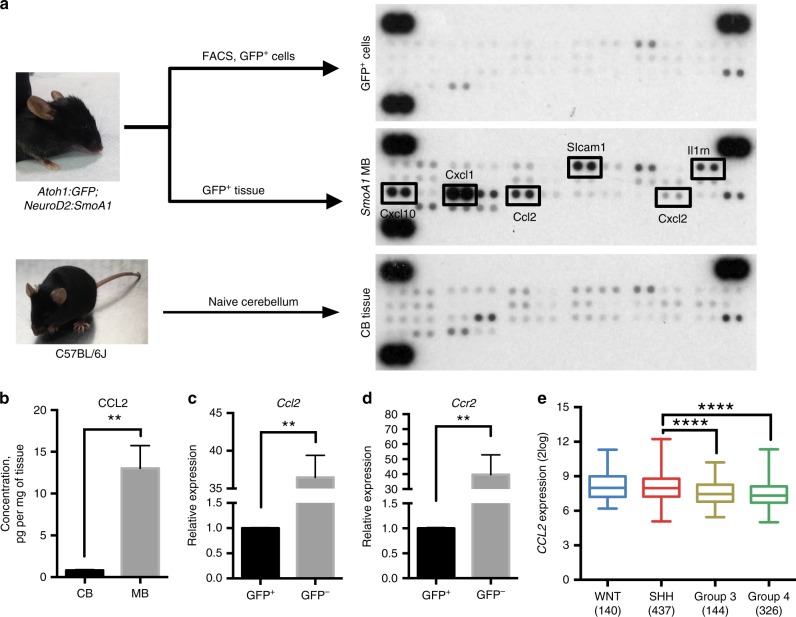
CCL2 is a key monocyte chemo-attractant cytokine in SHH MB. **a** Schematic of the experiment and results of cytokine analysis. *NeuroD2:SmoA1*;*Atoh1:GFP* tumour GFP tissue was extracted under a fluorescence microscope and either dissociated and FACS-sorted to isolate GFP^+^ cells or homogenised (*SmoA1* MB); naive cerebellum was used as a control (CB tissue). Images are representative of three independent experiments. **b** ELISA analysis of CCL2 cytokine level in cerebellum (CB) and medulloblastoma (MB) tissue, *N* = 9, Mann–Whitney *U* test, **P < 0.01. **c**, **d** Relative *Ccl2* (**c**) and *Ccr2* (**d**) expression levels in FACS-sorted cells, GFP^+^ are tumour cells, GFP^−^ are cells of the tumour microenvironment. *N* = 6, Mann–Whitney *U* test, **P < 0.01. **e** Microarray data analysis of human patient cohort for *CCL2* gene^13^. WNT Wingless, SHH Sonic Hedgehog, *N* = 140 for WNT, *N* = 437 for SHH, *N* = 144 for group 3, and *N* = 326 for group 4. Box plots represent median and quratiles with whiskers indicating range of values. One-way ANOVA with multiple comparisons, *F* = 19.17, ****P < 0.0001. Data are represented as mean ± S.E.M. for **b**–**d**

To investigate what drives monocytes and macrophages to the tumour, we performed cytokine arrays of sorted *SmoA1:GFP* medulloblastoma cells, medulloblastoma tumour tissue and normal cerebellum tissue. We found that several neutrophil-attractant molecules and the monocyte chemo-attractant protein 1 (MCP1, CCL2) (Fig. 3a) were highly expressed in tumours. CCL2 has been shown to be an important chemokine that attracts BM-derived myeloid cells into other types of brain tumours^15,17^. Using ELISA, we confirmed elevated levels of CCL2 in medulloblastoma tissue compared to normal cerebellum (Fig. 3b). PCR analysis showed that the cells of the TME produced more than 30-fold higher *Ccl2* and corresponding *Ccr2* genes when compared to tumour cells (Fig. 3c, d). Mining published microarray data^13,18^, we found a significant increase of *CCL2* expression in human SHH medulloblastomas, which further supports our murine model (Fig. 3e). Therefore, we decided to test whether deletion of *Ccl2* from the TME would result in a lower number of macrophages in tumours. We generated secondary tumours in *Ccl2*^−/−^ mice and found that the number of infiltrating myeloid cells in tumours was unchanged, with no difference in animal survival (Supplementary Fig. 5).

### Deletion of *Ccr2* from TME prevents immune-cell infiltration

**Fig. 4:**
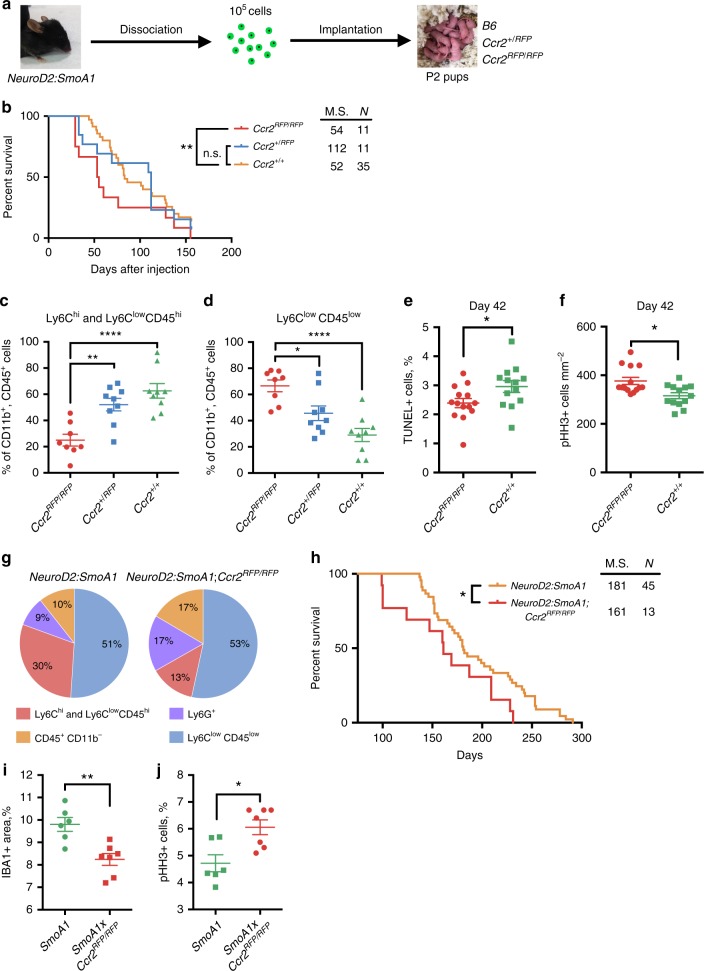
Reduction of myeloid TAMs significantly decreases animal survival. **a** Schematic of animal experiments. *NeuroD2:SmoA1* tumour tissue was extracted, dissociated into a single-cell suspension, and orthotopically injected into P2 pups of various genotypes. **b** Kaplan–Meier graphs of transgenic mice injected with *SmoA1* tumours, *Ccr2*^*RFP/RFP*^ (*N* = 11, red), *Ccr2*^*+/RFP*^ (*N* = 11, blue), and *Ccr2*^*+/+*^ (*N* = 35, gold), M.S. median survival. Log-rank Mantel–Cox test, **P < 0.01. Data represent three independent experiments. **c** Flow cytometry quantification of Ly6C^hi^ and Ly6C^low^CD45^hi^ (monocytes and macrophages), and **d** Ly6C^low^CD45^low^ (microglia) immune cells in tumours, *N* = 9 for *Ccr2*^*+/+*^ and *Ccr2*^*+/RFP*^, *N* = 8 for *Ccr2*^*RFP/RFP*^, one-way ANOVA with multiple comparisons, *F* = 14.4 (**c**), *F* = 13.21 (**d**), *P < 0.05, **P < 0.01, ****P < 0.0001. **e**, **f** Quantification of TUNEL^+^ cells (**e**) and pHH3^+^ cells (**f**) in tumours at 42nd day post-injection, *N* = 14 (*Ccr2*^*RFP/RFP*^), *N* = 13 (*Ccr2*^*+/+*^), Mann–Whitney *U* test, *P < 0.05. **g** Flow cytometry quantification of immune-cell composition in spontaneously occurring *NeuroD2:SmoA1* (*N* = 7, left graph) and *NeuroD2:SmoA1;Ccr2*^*RFP/RFP*^ (*N* = 7, right graph) murine tumour models. **h** Kaplan–Meier graphs of spontaneous tumours, *NeuroD2:SmoA1;Ccr2*^*RFP/RFP*^ (*N* = 13, red), *NeuroD2:SmoA1* (*N* = 45, gold), M.S. median survival. Log-rank Mantel–Cox test, *P < 0.05. **i**, **j** Quantification of IBA^+^ density (**i**) and percentage of pHH3^+^ cells (**j**) in *NeuroD2:SmoA1* (*SmoA1*, *N* = 6) and *NeuroD2:SmoA1;Ccr2*^*RFP/RFP*^ (*SmoA1*x*Ccr2*^*RFP/RFP*^, *N* = 7). Mann–Whitney *U* test, *P < 0.05, **P < 0.01. Data are represented as mean ± S.E.M. for **c**–**f**, **i**, **j**

Next, we showed that deletion of the corresponding receptor (*Ccr2*) from the host of allografted tumours decreased macrophage infiltration. We transplanted *SmoA1* CCR2^+/+^ tumours into *Ccr2* wild-type (+/+), heterozygous (+/RFP), and homozygous knock-in (RFP/RFP) mice (Fig. 4a). A Kaplan–Meier survival curve illustrated a significant decrease in median survival for *Ccr2*^*RFP/RFP*^ (53 days) mice bearing medulloblastoma compared to *Ccr2*^*+/+*^ mice (83 days; Fig. 4b). Using flow cytometry, we then quantified relative TAM composition in CD11b^+^CD45^+^ cells in tumours. We found a significant reduction in the percentage of mononuclear myeloid cells (monocytes and macrophages) from 63 ± 6 to 25 ± 4% (mean ± S.E.M., *N* = 9, Fig. 4c), and a corresponding increase in microglial cells from 18 ± 3 to 47 ± 6% (Fig. 4d) in *Ccr2*-deficient tumour-bearing mice.

We analysed tumours for apoptosis (TUNEL^+^, 7.3 ± 0.9 vs. 6.5 ± 1.1%, mean ± S.E.M., *N* = 4) and proliferation (phospho-H3^+^, 3.1 ± 0.2 vs. 3.5 ± 0.2%) levels in *Ccr2*^*RFP/RFP*^ vs. *Ccr2*^*+/+*^ mice at endpoint, but did not detect any significant difference between these groups (Supplementary Fig. 6). Therefore, we analysed tumours harvested on the 42nd day post-injection in both of these groups. We found that tumours injected in *Ccr2*^*RFP/RFP*^ mice had decreased apoptosis (TUNEL^+^, 2.4 ± 0.2% vs. 3 ± 0.2%, mean ± S.E.M., *N* = 13–14) and increased proliferation (phospho-histone H3^+^, 377 ± 15 vs. 316 ± 12 cell mm^−2^) compared to *Ccr2*^*+/+*^ mice (Fig. 4e, f). These data indicate that the reduced number of BMDMs is associated with reduced survival of medulloblastoma-bearing animals, decreased apoptosis, and increased proliferation in tumours.

A similar effect of *Ccr2* deletion was observed in spontaneous tumours in a *NeuroD2:SmoA1;Ccr2*^*RFP/RFP*^ murine model, where the loss of CCR2 receptor resulted in a decrease of total Ly6C^hi^ and Ly6C^low^CD45^hi^ cell number more than twofold, from 30% in *NeuroD2:SmoA1* down to 13% in *NeuroD2:SmoA1;Ccr2*^*RFP/RFP*^ (*N* = 7; Fig. 4g). The major decrease in immune microenvironment composition came from a reduction of infiltrating inflammatory monocytes, whose share decreased from 21% down to 2%. Analysis of survival curves for spontaneous *NeuroD2:SmoA1* (*N* = 45) and *NeuroD2:SmoA1;Ccr2*^*RFP/RFP*^ (*N* = 13) models revealed a significant decrease of median survival from 181 to 161 days for mice lacking the *Ccr2* gene (Fig. 4h). Immunohistochemistry analysis of tumour samples at the endpoint revealed a decrease in macrophage density from 9.8 ± 0.3% to 8.2 ± 0.2% and an increase in phospho-histone H3-positive cells from 4.7 ± 0.3% to 6.1 ± 0.3% in *NeuroD2:SmoA1* and *NeuroD2:SmoA1;Ccr2*^*RFP/RFP*^ mice, respectively (mean ± S.E.M., *N* = 6 and 7, respectively; Fig. 4i, j). These data confirm that a reduction in myeloid cell infiltration leads to significantly worse outcome for the animals, suggesting that these cells have an anti-tumoural role.

### BMDMs and microglia promote tumour cell death ex vivo

**Fig. 5:**
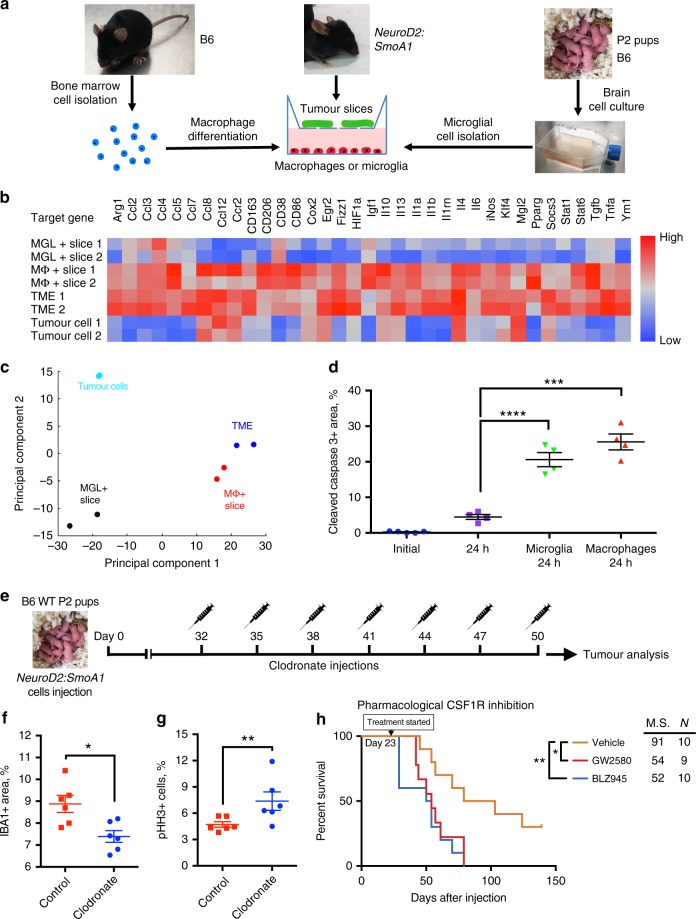
Cultured macrophages and microglial cells lead to tumour cell death ex vivo. **a** Schematic of experiment: bone marrow cells were extracted from control B6 mice and allowed to differentiate in culture for 6 days prior to exposure to organotypic medulloblastoma tumour slices. Loosely adherent microglial cells were isolated from brain cell culture mixture after culturing for 10 days in vitro. **b** qPCR analysis of pro- and anti-inflammatory macrophage-associated genes from FACS-sorted GFP^+^ tumour cells and GFP^−^ cells of TME compared to microglial cells (MGL) or macrophages (MΦ) exposed to tumour slices for 24 h. Two representative samples of 3–4 independent experiments for each group are shown. **c** 2D principal component analysis of PCR gene panel shown in **b**, TME (blue) – tumour microenvironment, MGL + Slice (black) – microglial cells exposed to tumour slice, MΦ + Slice (red) – macrophages exposed to tumour slices, FACS-sorted tumour cells shown in cyan. **d** Quantification of cleaved caspase 3^+^ area in tumour slices after exposure to BMDMs for 24 h in ex vivo conditions, *N* = 4 for each group, one-way ANOVA with multiple comparisons, *F* = 75.23, ****P < 0.0001. **e** Schematic of clodronate liposome treatment experiment. **f** Semi-quantitative analysis of macrophage linear density by measuring IBA1 staining in clodronate-treated and control tumours; each point represent analysis of at least five images (×20 magnification) from individual animals, *N* = 6, Mann–Whitney *U* test, **P < 0.01. **g** Quantification of pHH3^+^ cells in tumours after clodronate or control treatment, *N* = 6 for each group, Mann–Whitney *U* test, **P < 0.01. **h** Kaplan–Meier graphs of orthotopically implanted *NeuroD2:SmoA1* tumours, vehicle-treated (*N* = 10, gold), treated by daily oral gavage starting from day 23 post-injection with BLZ945 (*N* = 10, blue), or with GW2580 (*N* = 9, red). M.S. median survival. Data represent two independent experiments. Log-rank Mantel–Cox test, *P < 0.05, **P < 0.01. Data are represented as mean ± S.E.M. for **d**, **f**, **g**

We have designed a novel platform for investigating the effects of macrophages or microglial cells on tumour tissue in ex vivo conditions. A schematic of the setup and experimental design is shown in Fig. 5a. To determine the activation status of macrophages, we utilised a panel of pro- and anti-inflammatory genes that were used to determine macrophage phenotype in previous studies^19,20^. We used principal component analysis to evaluate macrophage gene expression profiles (Fig. 5b, Supplementary Fig. 7) and found that macrophages from co-culture are very similar to those sorted ex vivo from medulloblastoma tumours, but greatly differ from sorted tumour cells or cultured microglial cells exposed to tumour slices (Fig. 5c). Therefore, we used this system to further investigate the effects of macrophages and microglial cells on tumour slices ex vivo. We analysed tumour cell death by staining for cleaved caspase 3 and found a higher percentage of dying cells in tumour slices after 24-h exposure to either microglial cells (20.6 ± 1.8%, mean ± S.E.M., *N* = 4) or macrophages (25.6 ± 2.2%) compared to control samples (4.5 ± 0.7%) (Fig. 5d). At the same time, macrophage presence did not affect the number of proliferating cells in corresponding samples (Supplementary Fig. 8). Overall, these experiments indicate that macrophages have anti-tumoural properties in our ex vivo testing platform, and could potentially represent the situation in medulloblastoma tumours.

### Macrophage abrogation decreases animal survival

To confirm our findings obtained in the ex vivo experiments, we decided to use alternative methods to attempt to reduce macrophage number in tumours by using clodronate liposomes or inhibition of CSF1R. Clodronate liposomes induce apoptosis of professional phagocytes in murine models upon repeated administration^21^. Tumours were implanted in P2 pups as described and intravenous injection of liposomes began at day 32 post-implantation and was repeated every 3rd day. After 7 injections, mice were killed and tumour tissues were analysed. A schematic of the experiment is shown in Fig. 5e. We found that inhibition of macrophages with clodronate liposomes led to decreased TAM density (measured as IBA1^+^ area) from 8.9 ± 0.4 to 7.4 ± 0.3% (mean ± S.E.M., *N* = 6; Fig. 5f) and increased proliferation of tumour cells from 4.7 ± 0.3 to 7.4 ± 1% (Fig. 5g). Another pharmacological approach is to utilise the alternative macrophage/monocyte inhibition/repolarisation strategy of CSF1R inhibition using GW2580 and BLZ945 (refs. ^7,22^). P2 pups were orthotopically implanted with tumours and drugs were administered through a daily oral gavage routine starting at day 23 post-implantation. We found a significant decrease in median survival of mice that were treated with either drug (52 days for BLZ945, *N* = 10, and 54 days for GW2580, *N* = 9) compared to control vehicle-treated mice (91 days, *N* = 10) (Fig. 5h). These experiments further strengthen our finding that a reduction or repolarisation of TAMs in the TME leads to accelerated tumour progression.

## Discussion

Recent studies using murine models of glioblastoma demonstrated that increased infiltration of CCR2-positive inflammatory monocytes is associated with decreased survival time of tumour-bearing mice^23^. These studies also demonstrate that most TAMs in glioblastoma infiltrate the tumour bed early in tumour development and are crucial for malignant outgrowth^15,17^. Therefore, we set out to determine the role of TAMs in the paediatric high-grade medulloblastoma. Our results analysing RNA expression data in human medulloblastoma demonstrate enrichment of TAM-associated genes in the SHH subgroup. Similarly, recent studies showed that the highest number of TAMs and associated proteins are present in the same subgroup^11,12^. Consequently, we wished to investigate the functional role of TAMs in a murine model of SHH medulloblastoma, which is strikingly similar to human medulloblastoma at the gross pathology and molecular level. For the first time, we have characterised the immune TME cell composition of *NeuroD2:SmoA1* murine SHH medulloblastoma and found that it predominantly consists of CD45^hi^CD11b^+^ BM-derived myeloid cells and CD45^low^ microglial cells, with small fractions of CD45^+^Ly6G^+^ granulocytic cells and CD45^+^CD11b^−^ lymphocytes. Our results demonstrate that murine SHH medulloblastoma exhibits an immune-cell composition similar to that found in human medulloblastoma^10,12^. By generating medulloblastoma in *Ccr2*^*+/RFP*^*Cx3cr1*^*+/GFP*^ double knock-in mice we verified that, similar to glioblastoma, TAMs are a mixed population composed of BM-derived monocytes and macrophages (75%) and resident brain microglia (20%).

Previously, it has been shown that inflammatory monocytes promote gliomagenesis^23^. To define the role of inflammatory monocytes in medulloblastoma, we investigated whether deletion of *Ccl2* from TME will affects macrophage infiltration, but we found that their numbers were unchanged (Supplementary Fig. 5). Alternative mechanisms for macrophage recruitment via the CCL8-CCR2 pathway were then considered. For our further experiments, we used CCR2 (the main cytokine receptor responsible for monocyte trafficking) RFP knock-in mice. Using an orthotopic implantation model, we found that macrophage infiltration in tumour tissues was significantly reduced and survival time of tumour-bearing mice was decreased upon *Ccr2* gene deletion. A similar effect on survival was observed in spontaneously occurring tumours in mice that lack the CCR2 receptor. Here, animals demonstrated shorter median survival and increased cell proliferation level, indicating more aggressive tumours. This conclusion is further supported by our ex vivo findings showing that exposure of medulloblastoma organotypic slices to BMDMs resulted in increased tumour cell apoptosis and reduced proliferation. At the same time, pharmacological depletion of monocytes in the blood circulation of mice with clodronate liposomes led to decreased density of TAMs in tumour tissue and a corresponding increase in tumour cell proliferation. Others have shown that an alternative approach for macrophage depletion using CSF1R inhibition failed to deplete TAMs, but resulted in reduction of glioma tumour burden^7^, likely through macrophage repolarisation. Similar to this study, we did not observe TAM depletion in medulloblastoma tumours after CSF1R inhibition. In contrast, however, our studies revealed the opposite effect of the drug: we observed shorter median survival time in CSF1R-inhibited groups compared to the control group. Collectively, these results indicate that BMDMs have an anti-tumoural role in the SHH subgroup of medulloblastoma.

**Fig. 6:**
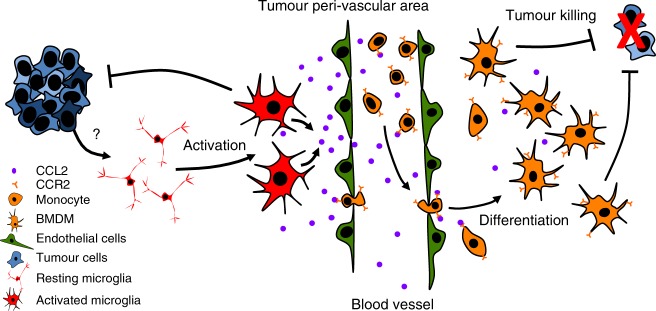
Proposed mechanism of macrophage recruitment to tumour tissue. Tumour presence activates microglia, which produce CCL2 and recruit monocytes from the blood stream. Monocytes infiltrate tumour tissues, differentiate into macrophages, and suppress tumour cells by promoting their death

A proposed schematic of macrophage recruitment and action is provided in Fig. 6. Monocytes from the blood stream are attracted to tumour tissue through a gradient of CCL2 cytokine released by the TME. Upon infiltration, monocytes differentiate and promote tumour cell death through an as-yet unknown mechanism. Contrary to the results in murine models of glioblastoma where BMDMs promote gliomagenesis, here we demonstrate that in murine models of SHH medulloblastoma, BMDMs promote tumour cell death. This intriguing difference raises the possibility that whether TAMs promote or inhibit tumour growth might be dependent upon tumour tissue origin. Several studies on TAMs in gliomas suggested that TAM-targeting therapies are an attractive avenue of investigation and may be successful in combination with the standard of care^9^. Our data suggest caution with the use of TAM-targeting therapies in medulloblastoma, since their function is tumour context-dependent.

Investigation of the complex interactions between tumour cells and cells of the TME could lead to development of novel personalised treatments, targeting specific subpopulations of cells that have key roles in tumour progression. This study sets a foundation for further investigation of the immune TME in medulloblastoma and suggests possible mechanisms through which infiltrating macrophages are cytotoxic to tumour cells. The toxicity may occur either through secreted factors or through a novel method such as TAM-mediated astrocyte activation, which has recently been shown to be neurotoxic in stroke and neurological disease^24^. Taken together, our study clearly demonstrates an anti-tumoural role of BM-derived inflammatory monocytes in medulloblastoma. Further investigation of the TME and mechanisms of TAM recruitment have the potential to lead to development of novel treatment approaches that will utilise the immune system as a powerful ally in the fight against cancer.

## Methods

### Patient data analysis

Raw gene expression data were obtained using GeneChip Human Genome U133 Plus 2.0 Array or Gene 1.1 ST Array from Affymetrix. All microarray data were collected from published datasets^13,25–29^. Transcriptome analyses were performed using R system software packages. Raw gene expression data from Affymetrix CEL files were normalised using MAS5 algorithm from the “affy” R package, and the resulting values were 2 log transformed. Normalised expression values of genes of interest were compared across medulloblastoma subgroups using the Student’s *t*-test and adjusted for multiple testing using the BH procedure. Human data were analysed using R2 genomics analysis and visualisation platform (https://r2.amc.nl). A Kaplan–Meier curve was plotted using available survival data from the Cavalli et al.^13^ dataset, with the survival cut-off chosen at the 10-year time point. Separation of patients by gene expression level was performed using either median or bottom quartile range.

### Animal experiments

Procedures involving animals were carried out in compliance with all relevant ethical regulations for animal testing and research. The protocol was approved by the Emory University Institutional Animal Care and Use Committee. C57BL/6J (B6) and *NeuroD2:SmoA1* mice were purchased from Jackson Laboratory. *NeuroD2:SmoA1* (ref. ^5^) and *Atoh1:GFP*^30^ mice were crossed to generate *NeuroD2:SmoA1;Atoh1:GFP*, in which all tumour cells produce green fluorescent protein (GFP). We also generated crosses of *NeuroD2:SmoA1;Ccr2*^*RFP/RFP*^ mice. *Cx3cr1*^*+/GFP*^*;Ccr2*^*+/RFP*^ were used to study immune-cell lineage, as these mice express red fluorescent protein (RFP) in CCR2-positive peripheral monocytes, T-cells and NK cells, and express GFP in monocytes, dendritic cells, NK cells and brain microglia^31^. Animals were provided food and water ad libitum and maintained with an alternating 12-hour light and dark cycle. The tumour-bearing *SmoA1* mice used in these studies were predominantly females within an age range of 3–6 months. Secondary medulloblastoma tumours were generated by orthotopic allografts of 10^5^ freshly dissociated *SmoA1* tumour cells (that included cells of the microenvironment) into postnatal day 0 (P0)-P2 pups of different genotypes (*B6, Cx3cr1*^*+/GFP*^*;Ccr2*^*+/RFP*^*, Ccl2*^+/−^*, Ccl2*^−/−^*, Ccr2*^*+/RFP*^*, Ccr2*^*RFP/RFP*^).

To pharmacologically inhibit macrophages, we utilised two alternative approaches: using clodronate liposomes or CSF1R inhibition. Clodronate and control liposomes were purchased from clodronate liposomes.com and used according to the manufacturer’s guidelines. Clodronate liposomes were injected intravenously with a single dose of 100 µl per animal, every 72 h. To inhibit CSF1R, we utilised two inhibitors, GW2580 (LC Laboratories) and BLZ945 (Selleckchem), which previously shown to prolong survival of glioma-bearing mice^7,22^. Although the drugs do not decrease the number of TAMs, they change their polarisation from M2 to M1^7,22^. We utilised a dose of 160 mg kg^−1^ for GW2580 and 200 mg kg^−1^ for BLZ945 formulated in 5% dimethyl sulfoxide, 35% polyethylene glycol 400, and 60% phosal as a daily oral gavage vehicle. To generate tumours, animals were orthotopically injected with freshly dissociated, single-cell suspension from two independent *NeuroD2:SmoA1* tumours and were randomly allocated to a treatment or control group.

### Cell cultures

Bone marrow-derived monocytes were isolated from C57BL/6J mice 4–8 weeks of age using an established procedure^32^. Briefly, bone marrow cells were flushed from extracted tibiae and femurs using harvest solution (PBS with 0.02% Bovine Serum Albumin (STEMCELL), 1 U ml^−1^ Heparin (STEMCELL), 300 U ml^−1^ DNase (Worthington)). Homogenised bone marrow suspension was strained through 40-µm mesh and plated on non-tissue culture-treated 15 cm petri dishes. Macrophages were allowed to differentiate for a period of 6 days in DMEM medium supplemented with 10% Fetal Bovine Serum (FBS, Gibco), 1% penicillin-streptomycin (Gibco), and 40 ng ml^−1^ of recombinant murine CSF1 (Biolegend) before passaging. Macrophages were seeded at density of 1.5 × 10^6^ per well of a six-well plate for all experiments.

Microglial cells were isolated from P2 C57BL/6J mouse pups using a previously established procedure^33^. Briefly, murine brains were dissected out and meninges were removed under a microscope. Brains were dissociated using 0.25% trypsin with DNase and the resulting single-cell suspension was strained through 40-µm strainers. Cells were seeded in poly-d-lysine-coated T75 flasks, grown in the same medium as BMDMs, and cultured for 10 days, and then collected by vigorous shaking. Microglial cells were seeded at a density of 8 × 10^5^ per well of a six-well plate for all experiments.

### Ex vivo slice cultures

Medulloblastoma primary tumour slices were isolated from *SmoA1* tumour-bearing mice and cultured similar to a previous report^34^. Briefly, whole cerebellum containing tumour was extracted and embedded in 4% low-melt agarose at 37–40 °C. Extracted samples were sliced in ice-cold sterile phosphate buffered saline (PBS) using a Leica V1200S vibratome with amplitude 1.5 mm, speed of 0.3 mm s^−1^, and 300 µm-thick slices. Tumour tissue slices were transferred to inserts in 6-well plates with fresh medium (Neurobasal (Gibco) medium supplemented with B27 supplement and 1% penicillin-streptomycin) and incubated at 37 °C overnight prior to exposure to macrophages.

### RNA extraction and RT-PCR

Tissue or cells samples were homogenised in Trizol (ThermoFisher) reagent, and stored frozen at −80 °C for up to 2 weeks. Upon phase separation using chloroform and subsequent centrifugation, aqueous phase was mixed with ethanol to 35% and transferred to RNeasy mini kit columns (QIAGEN), followed by washing according to the manufacturer’s suggested procedure. cDNA synthesis was performed using a High-Capacity cDNA Reverse Transcription Kit (Applied Biosystems) according to the manufacturer’s protocol. Quantitative real-time PCR was performed using Sso Advanced Universal SYBR Green Supermix (Biorad) in a CFX96 real-time PCR detection system. A list of primers used can be found in Supplementary Table 1.

### Immunohistochemistry and immunofluorescence

Immunohistochemistry on paraffin-embedded and sectioned brain samples was performed using a standard procedure. Briefly, slides were deparaffinised and rehydrated. Antigen retrieval was performed using low-pH antigen-unmasking solution (Vector). Tissue was then permeabilised and peroxidases were blocked in 0.3% hydrogen peroxide. Slides were incubated with blocking buffer (PBS, 0.1% Triton X-100, 5% goat serum) for 1 h at room temperature and then incubated with primary antibody overnight at 4 °C. After washing, slides were exposed to biotinylated secondary antibodies (Vector, #BA-1100 and #BA-9401, 1:200) for 1 h at room temperature, washed again, and then exposed to Elite ABC reagent mixture (Vector) for 1 h at room temperature. Staining was performed using DAB peroxidase substrate kit (Vector) and was followed by counterstaining with haematoxylin. Slides were scanned using the whole-slide scanner Hamamatsu Nanozoomer 2.0HT at ×40 magnification and images were analysed using NDP.view2, Cell Profiler, and FIJI (Image J, NIH). Immunofluorescence on frozen and paraffin-embedded tissues was performed using a similar protocol as the immunohistochemistry, but using donkey serum as a blocker and fluorescently labelled donkey secondary antibodies (1:500, ##A32766, A21207, A21208, Invitrogen) and DAPI as a counterstain. Imaging was performed using an upright Leica DM2500 microscope equipped with a DFC365FX camera and Leica software. Confocal analysis was performed with an inverted Olympus FV1000 microscope. The list of antibodies for immunofluorescence and immunohistochemistry used in the present work includes: anti-phospho-histone H3 (Cell Signalling Technology, #9701, 1:200), anti-IBA1 (WAKO, 019-19741, 1:200), anti-CD31 (Dianova, DIA-310, 1:50), and anti-RFP (Rockland, 600-401-379, 1:200).

### Cytokine array and other assays

Mouse Cytokine array panel A (ARY006, R&D systems) was used to measure cytokine levels in various cells and tissues using the manufacturer’s provided protocol. Tumour tissue was extracted from *NeuroD2:SmoA1;Atoh1:GFP* mice and dissociated using papain digestion. Approximately 20 million GFP^+^ cells were sorted in PBS, centrifuged at 350 × *g* for 5 min, and lysed in the provided buffer. CCL2 cytokine content in tissue lysates was performed using R&D systems kit DY479-05 according to the manufacturer’s instructions. TUNEL apoptosis assays were performed on paraffin-embedded tumour tissue sections using in situ cell death detection kits (Roche Diagnostics, #11684795910).

### Flow cytometry and fluorescence-activated cell sorting

Isolated brain tumour tissue was digested in cysteine-activated papain (Worthington, 50 U ml^−1^ in HBSS, pH 7) with 300 U ml^−1^ DNAse for 5 min at 37 °C, triturated, and strained through a 70 µm strainer. Cell suspension was resuspended in HBSS and subsequently underlayed with 35 and 70% Percoll (GE Healthcare) and centrifuged for 15 min at 800 × *g*. Interphase between layers was collected and washed using PBS with 1% bovine serum albumin and 0.1% NaN_3_. After Fc receptor blocking (TruStain FcX™ (Clone 93) #101320, BioLegend), cells were incubated with fluorochrome-conjugated primary antibodies at 1:100 dilution. Antibodies for flow cytometry (V450-Ly6G, #560603; PE-Cy7-Ly6C #560593; PerCP-Cy™5.5-CD11b #550993; and APC-CD45 #559864) were purchased from BD Biosciences. The gating strategy is depicted in Supplementary Fig. 1: we selected CD11b^hi^CD45^+^ population and separated Ly6G^+^ neutrophils and Ly6C^+^ newly infiltrated monocytes. The CD45^low^Ly6C^low^ population is microglial cells and the CD45^hi^Ly6C^low^ population is cells of myeloid origin, differentiated TAMs. Analysis was performed using CytoFLEX platform (Beckman Coulter) with CytExpert software. Analysis of data was performed using FlowJo software. Fluorescence-activated cell sorting was performed using Sony SH800 cell sorter to collect GFP^+^ and GFP^−^ populations from *NeuroD2:SmoA1;Atoh1:GFP* tumours.

### Statistical analysis

Statistical differences were calculated using GraphPad Prism software using an unpaired, nonparametric Mann–Whitney *U* test when comparing ranks of two groups and a one-way ANOVA followed by Kruskal–Wallis unpaired nonparametric test with multiple comparisons when comparing three or more groups. Measurements were taken from distinct samples, i.e., biological replicates. PCR data were analysed using principal component analysis in MATLAB software, and an expression cell plot was generated using JMP pro 13 software.

### Reporting summary

Further information on research design is available in the Nature Research Reporting Summary linked to this article.

## Supplementary information

Supplementary Information

Reporting Summary

## Data Availability

The gene expression data from Cavalli et al.^13^ used in this study are available in GEO under the accession numbers GSE85218, GSE37382 and GSE37384. All transcriptional data is publically available and can be freely explored using the R2 data portal (https://hgserver1.amc.nl/cgi-bin/r2/main.cgi?&dscope=MB500&option=about_dscope). All other data supporting the findings from this study are available within the article and Supplementary Information Files and from the corresponding author upon reasonable request.
