# Supplementary material for: Tumour-associated macrophages exhibit anti-tumoural properties in Sonic Hedgehog medulloblastoma
Source: Nat Commun. 2019 Jun 3;10:2410. doi: 10.1038/s41467-019-10458-9 (PMC6546707; doi:10.1038/s41467-019-10458-9)
Supplement: Supplementary file 1 — Supplementary Information [file 41467_2019_10458_MOESM1_ESM.pdf]

Tumour-Associated Macrophages Exhibit Anti-Tumoural Properties in Sonic Hedgehog Medulloblastoma

Maximov et al.

Supplementary Information

Supplementary Figure 1.

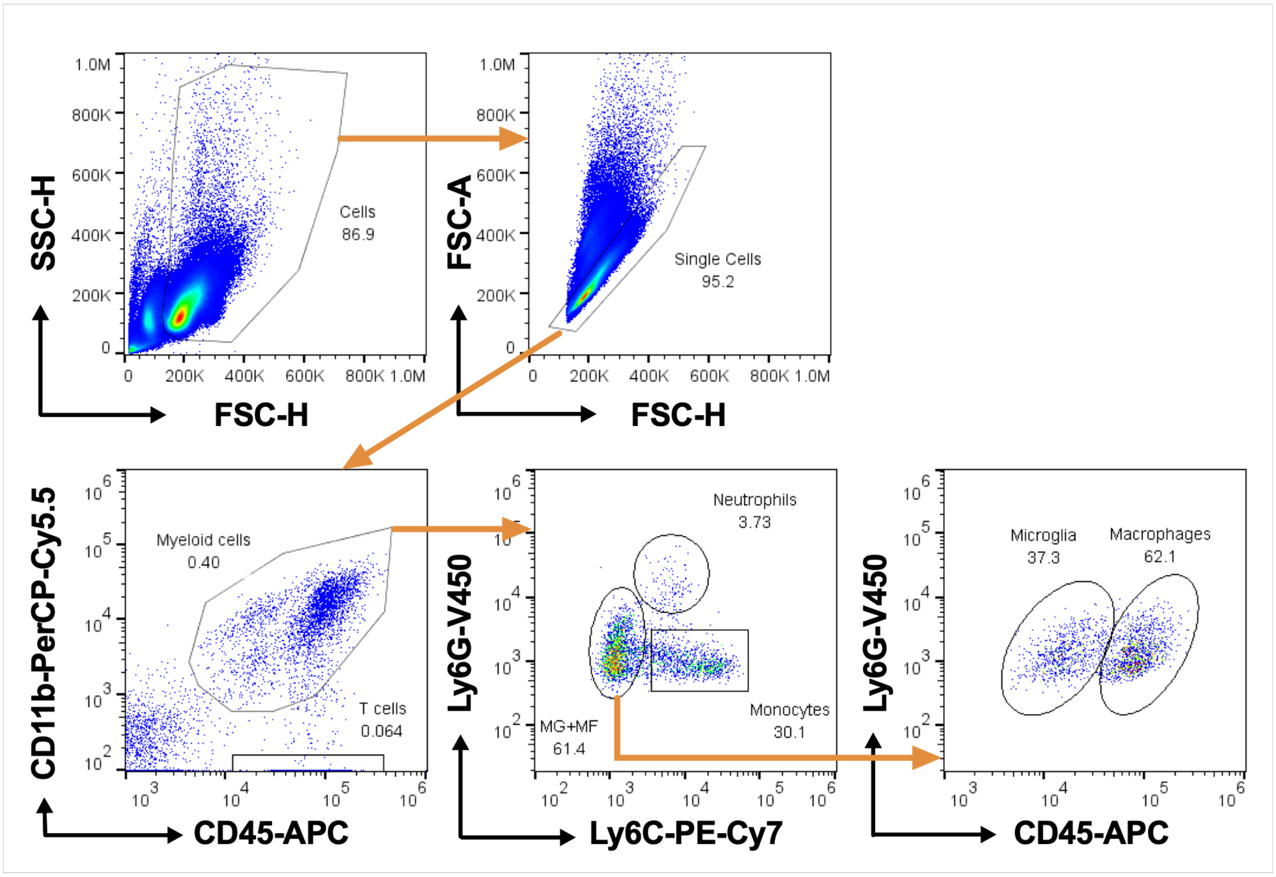

Supplementary Figure 1. Gating strategy to separate immune cells populations. After selection of cell population, we separated single cells, followed by selection of CD11b and CD45 positive cells. $CD45^{+}CD11b^{-}$ cells were considered as lymphoid cells. $CD45^{+}$ $CD11b^{+}$ cells were a mix of bone marrow-derived myeloid cells and microglia. We separated them by Ly6G and Ly6C expression – $Ly6G^{+}$ cells are neutrophils, $Ly6C^{+}$ cells are freshly infiltrated monocytes. Ly6C low cells were separated into $CD45^{low}$ (microglia) and $CD45^{hi}$ (macrophage) cells.

Supplementary Figure 2.

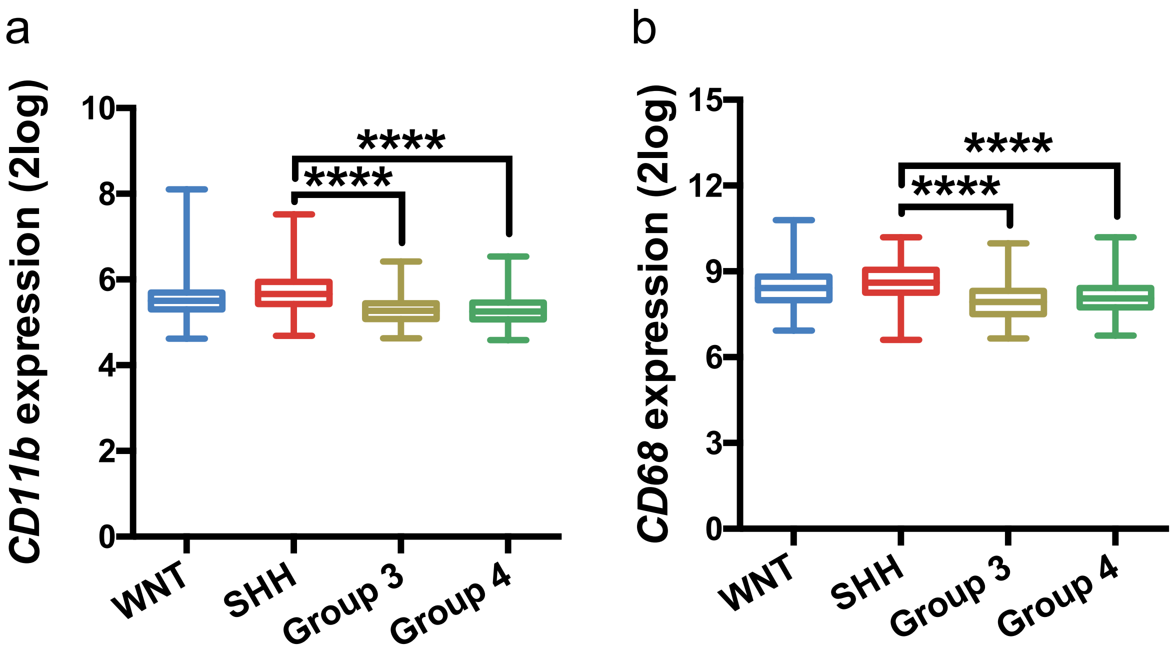

Supplementary Figure 2. SHH medulloblastoma has pronounced activation of macrophage-associated genes. Microarray data analysis of human patients MB cohort for macrophage-related genes *CD11b* (a), *CD68* (b) from previously published study[15]. WNT – Wingless, SHH – Sonic Hedgehog, N=70 (WNT), N=223 (SHH), N=144 (Group 3), N=326 (group 4). One way ANOVA with multiple comparisons. ****P<0.0001 (F= 65.45 (a), 54.94 (b)).

Supplementary Figure 3.

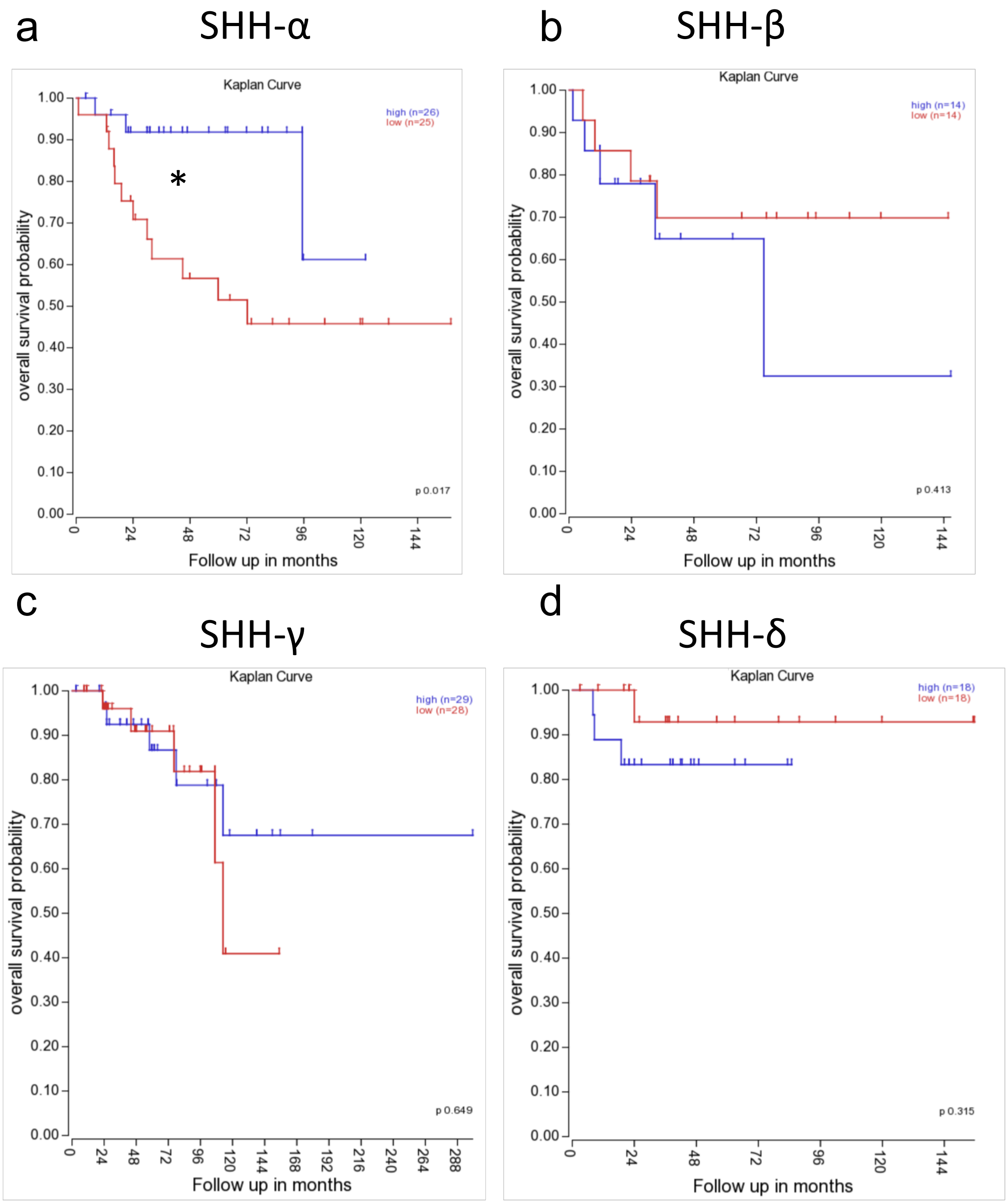

Supplementary Figure 3. SHH medulloblastoma α subtype patients have the highest survival benefit from presence of macrophages. Kaplan curves for MB patients separated by SHH subgroup and differentiated by macrophage gene *AIF1* expression. Log-rank Mantel-Cox test, *P<0.05.

Supplementary Figure 4.

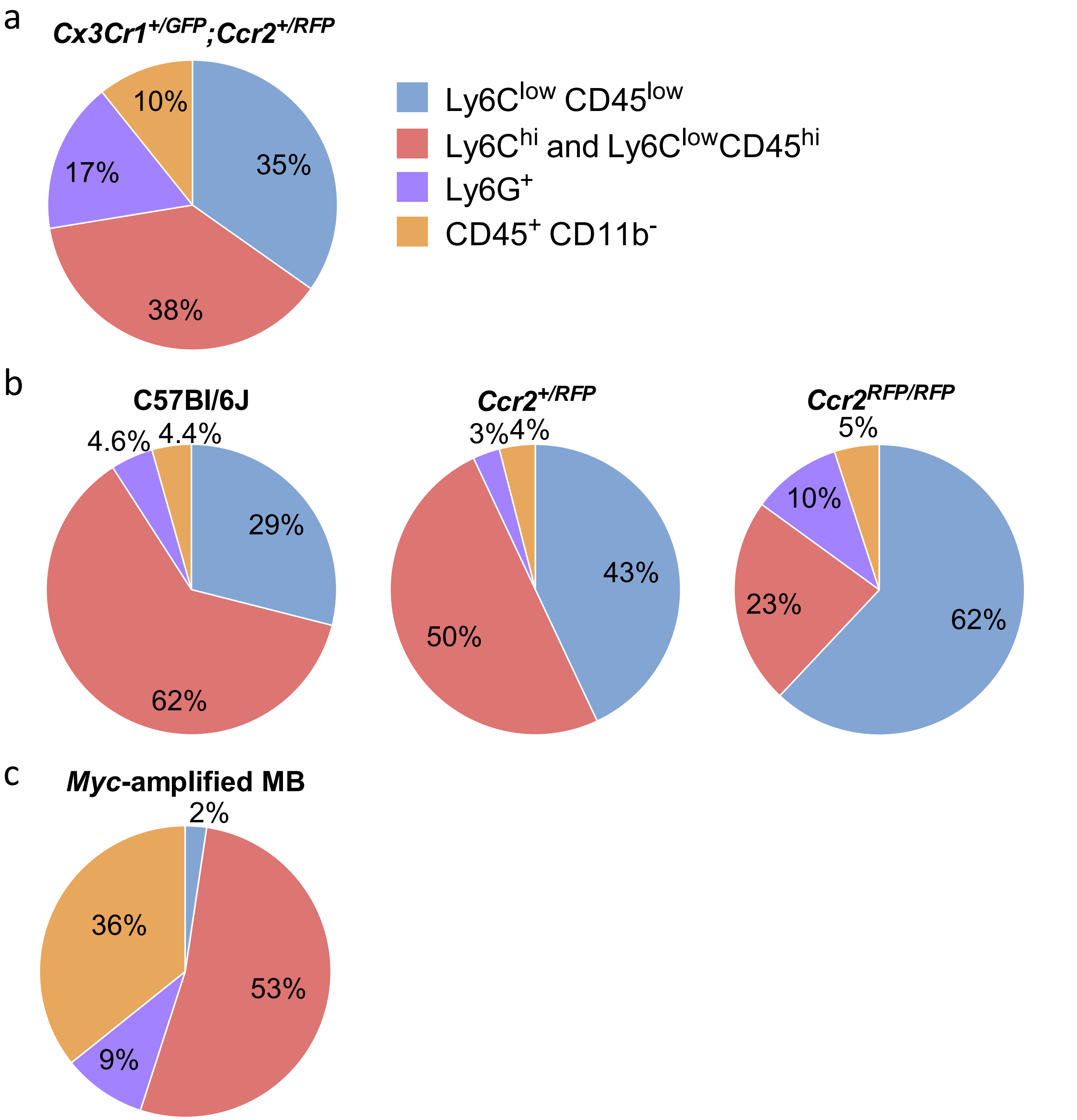

Supplementary Figure 4. Deletion of *Ccr2* from tumour microenvironment decreases infiltration of macrophages in orthotopic implantation model. A) Immune cell composition of *NeuroD2:SmoA1* tumours orthotopically implanted in *Ccr2*$^{+/RFP}$*Cx3cr1*$^{+/GFP}$ mice for immune cells tracing, N=5. B) *NeuroD2:SmoA1* tumors orthotopically implanted in C57Bl/6J, N=9; *Ccr2*$^{+/RFP}$, N=9; *Ccr2*$^{RFP/RFP}$, N=7. C) *Myc*-amplified MB (group 3) model implanted in B6 mice (N=4).

Supplementary Figure 5.

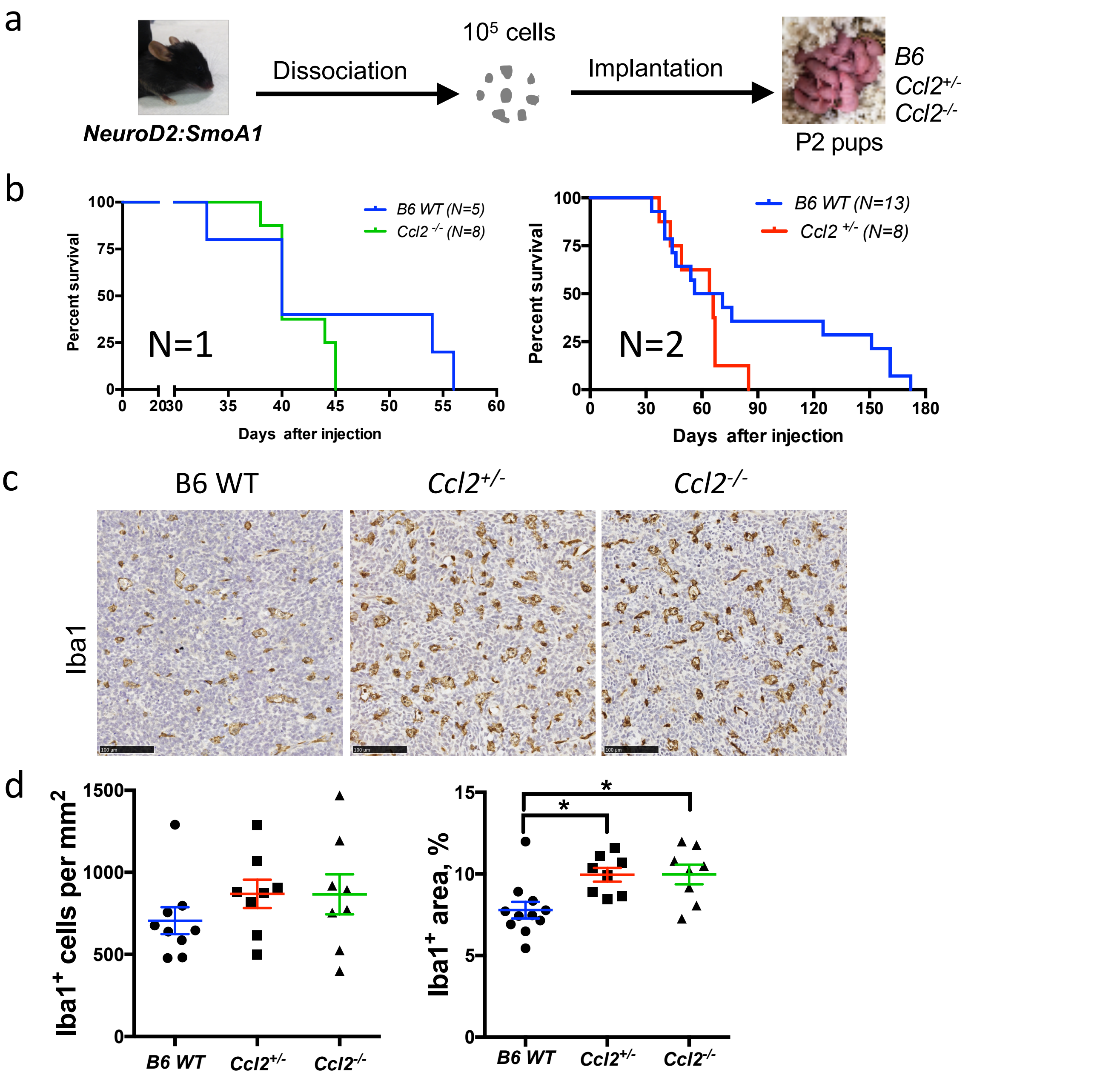

Supplementary Figure 5. Generation of MB tumours in *Ccl2*$^{-/-}$ mice does not decrease number of TAMs. A) Schematic of animal experiments. *NeuroD2:SmoA1* tumour tissue was extracted, dissociated into a single cell suspension and orthotopically injected into P2 pups of indicated genotypes. B) Kaplan-Meier graphs of genetically modified mice injected with *SmoA1* tumours, *Ccl2*$^{-/-}$ (N=8, green), *Ccl2*$^{+/-}$ (N=8, red), and *Ccl2*$^{+/+}$ (N=13, blue), N represents indicated number of independent tumour injection experiments. C) Representative IHC images for TAM marker Iba1 in generated tumours. D) Quantification of Iba1+ cells number and area in tumours at the endpoint, N=9 for *Ccl2*$^{+/+}$, N=8 for *Ccl2*$^{+/-}$ and *Ccl2*$^{-/-}$, Mean ± S.E.M. One-way ANOVA, *P<0.05.

Supplementary Figure 6.

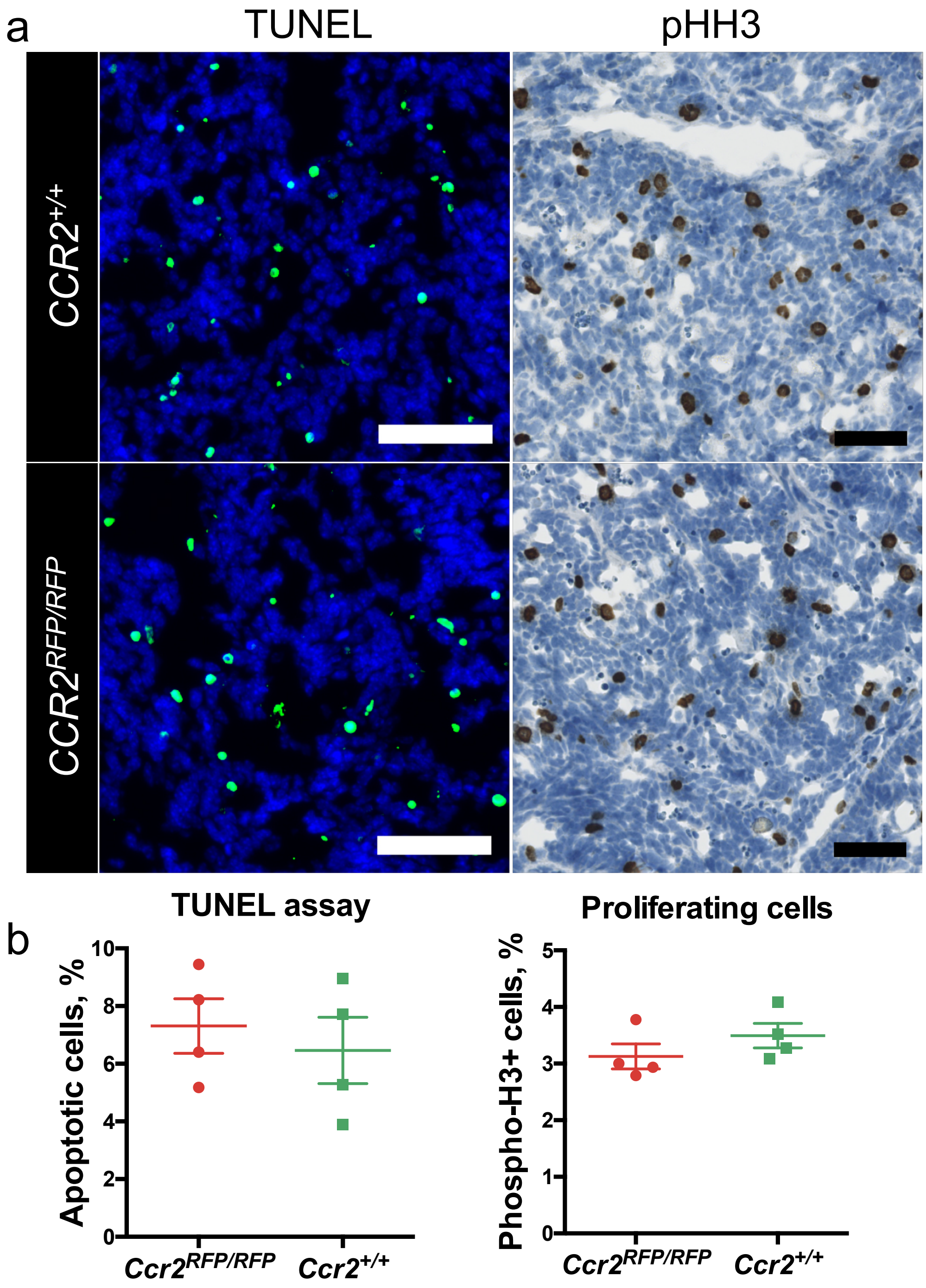

Supplementary Figure 6. Deletion of *Ccr2* from tumour microenvironment does not affect apoptosis or proliferation levels at the endpoint. A) TUNEL assay and phosphohistone-H3 immunohistochemistry analyses of tumours generated in *Ccr2*$^{+/+}$ and *Ccr2*$^{RFP/RFP}$ mice. Scale bars represents 50 μm; data are representative of at least four independent experiments. B) Quantification of TUNEL or phosphohistone-H3 positive cells in (A). Each data point is an average value of at least five images analyzed. Mean ± S.E.M. Mann-Whitney U test, $P>0.05$.

Supplementary Figure 7.

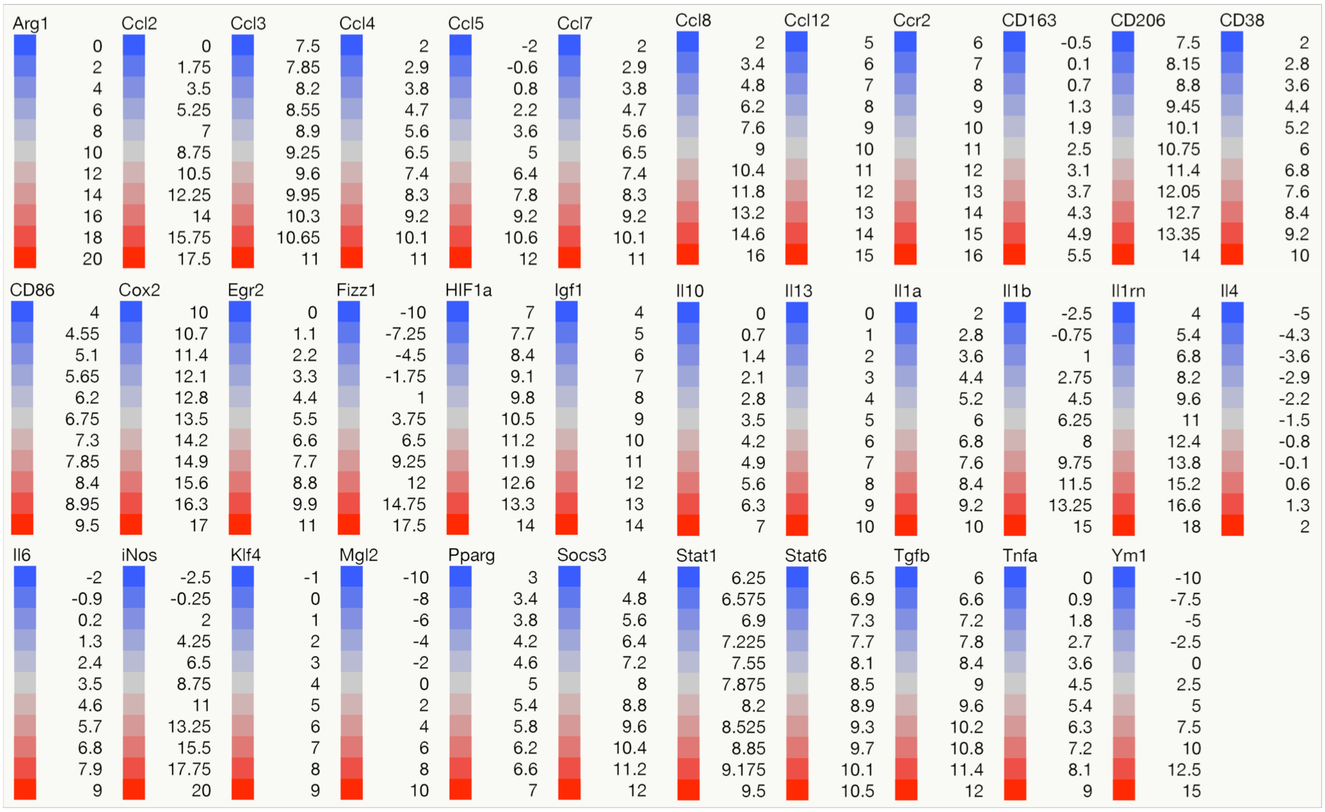

Supplementary Figure 7. Expression range (*log2* transformed) legends for microarray image shown in Fig. 5b.

Supplementary Figure 8.

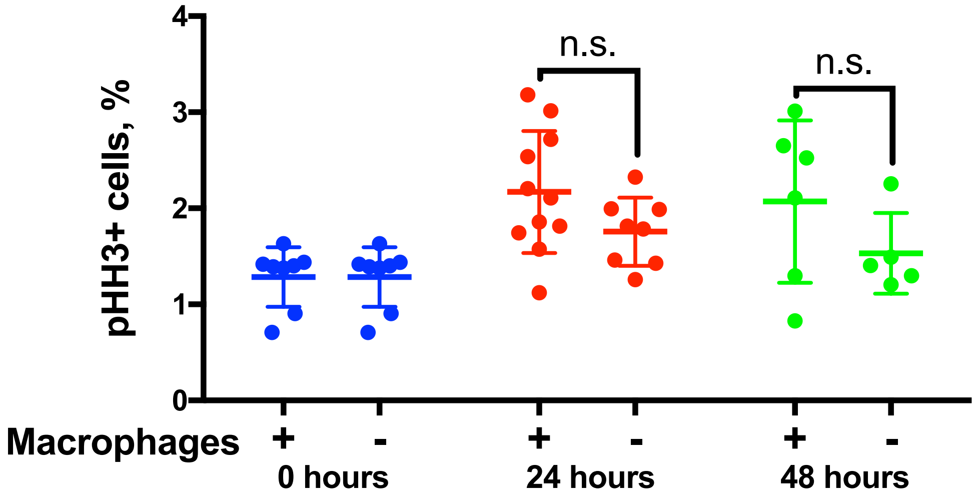

Supplementary Figure 8. Macrophages presence does not affect tumour cells proliferation. Tumour slices were exposed to cultured differentiated macrophages for up to 48 hours. Phopho histone-H3 IHC staining was quantified using whole slide scanning and image analysis as described in methods section. Mean ± S.E.M., Mann-Whitney U test, n.s. P>0.05.

Supplementary Table 1. List of PCR primers used in current work.

Target name	Forward primer 5'-3'	Reverse primer 5'-3'
Ccl2	CCCACTCACCTGCTGCTACT	TCTGGACCCATTCCTTCTTG
Actb	GGCTGTATTCCCCTCCATCG	CCAGTTGGTAACAATGCCATGT
SOCS3	TACTGAGCCGACCTCTCTC	AGCTGGGTCACTTTCTCATA
COX2	CCGTCCCCTCACTAGGACTT	ATCCTGGTCGGTTTGATGCT
CCL5	TGCCCACGTCAAGGAGTATTTC	AACCCACTTCTTCTCTGGGTTG
STAT1	TGGTGAAATTGCAAGAGCTG	CAGACTTCCGTTGGTGGATT
HIF1a	GGGTACAAGAAACCACCCAT	GAGGCTGTGTCGACTGAGAA
CCL3	ACTGCCTGCTGCTTCTCCTACA	AGGAAAATGACACCTGGCTGG
CCL4	ACCATGAAGCTCTGCGTGTC	CCATTGGTGCTGAGAACCCT
Cnn3	CATGACAGCCTATGGGACTC	CTCCCGAGGGTAGTCGTCTG
Il1a	AGTCAACTCATTGGCGCTTGA	AGAGAGATGGTCAATGGCAGA
CD38	GAAGACTACGCCCCACTTGT	ATGGGCCAGGTGTTTGGATT
IL10	GCTCTTACTGACTGGCATGAG	CGCAGCTCTAGGAGCATGTG
CD163	TGCTGTCACTAACGCTCCTG	TCATTCATGCTCCAGCCGTT
PPARg	GCCCTTTGGTGACTTTATGGA	GCAGCAGGTTGTCTTGGATG
STAT6	ACCTGTCCATTCGCTCACTG	CTCTGGAGTAGGAAGGGGCT
KLF4	GTGCCCCGACTAACCGTTG	GTCGTTGAACTCCTCGGTCT
IL4	AGATGGATGTGCCAAACGTCCTCA	AATATGCGAAGCACCTTGGAAGCC
IL13	GGCAGCATGGTATGGAGTGT	CTTGCGGTTACAGAGGCCAT
IGF1	AGACAGGCATTGTGGATGAG	TGAGTCTTGGGCATGTCAGT
EGR2	TGCTAGCCCTTTCCGTTGA	TCTTTTCCGCTGTCCTCGAT
MGL2	GATAACTGGCATGGACATATG	TTTCTAATCACCATAACACATTC
Il1b	CAGGCTCCGAGATGAACAAC	GGTGGAGAGCTTTCAGCTCATAT
iNOS	CCCTTCAATGGTTGGTACATGG	ACATTGATCTCCGTGACAGCC
Arg1	GGAATCTGCATGGGCAACCTGT	AGGGTCTACGTCTCGCAAGCCA
Fizz1	TCCCAGTGAATACTGATGAGA	CCACTCTGGATCTCCCAAGA
Ym1	CAGGTCTGGCAATTCTTCTGAA	GTCTTGCTCATGTGTGTAAGTGA
Il6	GCCTTCTTGGGACTGATGCT	AGTCTCCTCTCCGGACTTGTG
TNFa	AAGCCTGTAGCCCACGTCGTA	GGCACCACTAGTTGGTTGTCTTTG
CD86	TTGTGTGTGTTCTGGAAACGGAG	AACTTAGAGGCTGTGTTGCTGGG
TGFb	CTTCAATACGTCAGACATTCGGG	GTAACGCCAGGAATTGTTGCTA
CD206	GGCAGGATCTTGGCAACCTAGTA	CCTTTCTTCCGACTCTTCACCC
Ccr2	TGTGATTGACAAGCACTTAGACC	TGGAGAGATACCTTCGGAACTT
