# Supplementary material for: Tumour-associated macrophages exhibit anti-tumoural properties in Sonic Hedgehog medulloblastoma
Source: Nat Commun. 2019 Jun 3;10:2410. doi: 10.1038/s41467-019-10458-9 (PMC6546707; doi:10.1038/s41467-019-10458-9)
Supplement: Supplementary file 2 — Reporting Summary [file 41467_2019_10458_MOESM2_ESM.pdf]

nature research

Corresponding author(s): Anna M. Kenney

Last updated by author(s): Mar 28, 2019

Reporting Summary

Nature Research wishes to improve the reproducibility of the work that we publish. This form provides structure for consistency and transparency in reporting. For further information on Nature Research policies, see Authors & Referees and the Editorial Policy Checklist.

Statistics

For all statistical analyses, confirm that the following items are present in the figure legend, table legend, main text, or Methods section.

n/a	Confirmed	
☐	☒	The exact sample size (*n*) for each experimental group/condition, given as a discrete number and unit of measurement
☐	☒	A statement on whether measurements were taken from distinct samples or whether the same sample was measured repeatedly
☐	☒	The statistical test(s) used AND whether they are one- or two-sided<br>*Only common tests should be described solely by name; describe more complex techniques in the Methods section.*
☒	☐	A description of all covariates tested
☐	☒	A description of any assumptions or corrections, such as tests of normality and adjustment for multiple comparisons
☐	☒	A full description of the statistical parameters including central tendency (e.g. means) or other basic estimates (e.g. regression coefficient) AND variation (e.g. standard deviation) or associated estimates of uncertainty (e.g. confidence intervals)
☐	☒	For null hypothesis testing, the test statistic (e.g. *F*, *t*, *r*) with confidence intervals, effect sizes, degrees of freedom and *P* value noted<br>*Give P values as exact values whenever suitable.*
☒	☐	For Bayesian analysis, information on the choice of priors and Markov chain Monte Carlo settings
☒	☐	For hierarchical and complex designs, identification of the appropriate level for tests and full reporting of outcomes
☒	☐	Estimates of effect sizes (e.g. Cohen's *d*, Pearson's *r*), indicating how they were calculated

Our web collection on statistics for biologists contains articles on many of the points above.

Software and code

Policy information about availability of computer code

Data collection	N/A
Data analysis	N/A

For manuscripts utilizing custom algorithms or software that are central to the research but not yet described in published literature, software must be made available to editors/reviewers. We strongly encourage code deposition in a community repository (e.g. GitHub). See the Nature Research guidelines for submitting code & software for further information.

Data

Policy information about availability of data

All manuscripts must include a data availability statement. This statement should provide the following information, where applicable:
- Accession codes, unique identifiers, or web links for publicly available datasets
- A list of figures that have associated raw data
- A description of any restrictions on data availability

The data that support the findings of this study are available from the corresponding author upon reasonable request.

Field-specific reporting

Please select the one below that is the best fit for your research. If you are not sure, read the appropriate sections before making your selection.

☒ Life sciences ☐ Behavioural & social sciences ☐ Ecological, evolutionary & environmental sciences

For a reference copy of the document with all sections, see nature.com/documents/nr-reporting-summary-flat.pdf

Life sciences study design

All studies must disclose on these points even when the disclosure is negative.

Sample size	We chose sample sizes based on our previous experience with brain tumour survival curves. Collected sample sized clearly demonstrate statistical significance upon analysis.
Data exclusions	No data exclusion was carried out
Replication	We performed in vivo experiments several times where possible and results were reproducible
Randomization	samples were randomly distributed among groups
Blinding	Image analysis was performed in a blinded manner

Reporting for specific materials, systems and methods

We require information from authors about some types of materials, experimental systems and methods used in many studies. Here, indicate whether each material, system or method listed is relevant to your study. If you are not sure if a list item applies to your research, read the appropriate section before selecting a response.

Materials & experimental systems

n/a	Involved in the study	
☐	☒	Antibodies
☒	☐	Eukaryotic cell lines
☒	☐	Palaeontology
☐	☒	Animals and other organisms
☒	☐	Human research participants
☒	☐	Clinical data

Methods

n/a	Involved in the study	
☒	☐	ChIP-seq
☐	☒	Flow cytometry
☒	☐	MRI-based neuroimaging

Antibodies

Antibodies used	anti-phospho-histone H3 (Cell Signalling Technology, #9701, 1:200), anti-IBA1 (WAKO, 019-19741, 1:200), anti-CD31 (Dianova, DIA-310, 1:50), and anti-RFP (Rockland, 600-401-379, 1:200), biotinylated secondary antibodies (Vector, #BA-1100 and #BA-9401, 1:200), fluorescently-labelled donkey secondary antibodies (1:500, ##A32766, A21207, A21208, Invitrogen), Flow cytometry antibodies (V450-Ly6G, #560603; PE-Cy7-Ly6C #560593; PerCP-Cy™5.5-CD11b #550993; and APC-CD45 #559864 BD Biosciences).
Validation	Validation information and citations for antibodies could be found using these numbers: RRID:AB_331535, RRID:AB_839504, RRID:AB_2631039, RRID:AB_2209751, RRID:AB_1727564, RRID:AB_394628, RRID:AB_10515293, RRID:AB_398672 on the http://antibodyregistry.org website

Animals and other organisms

Policy information about studies involving animals; ARRIVE guidelines recommended for reporting animal research

Laboratory animals	Mice of C57BL6/J were used. We also utilized genetically modified animals of following genotypes: NeuroD2:SmoA1, Atoh1:GFP, NeuroD2:SmoA1;Atoh1:GFP, NeuroD2:SmoA1;Ccr2RFP/RFP, Cx3cr1+/GFP;Ccr2+/RFP, Ccl2+/-, Ccl2-/-, Ccr2+/RFP, Ccr2RFP/RFP.
Wild animals	This study did not involve wild animals
Field-collected samples	Samples were not collected in the field
Ethics oversight	Procedures involving animals were carried out in compliance with all relevant ethical regulations for animal testing and research. The protocol was approved by the Emory University Institutional Animal Care and Use Committee.

Note that full information on the approval of the study protocol must also be provided in the manuscript.

Flow Cytometry

Plots

Confirm that:

☒ The axis labels state the marker and fluorochrome used (e.g. CD4-FITC).

☒ The axis scales are clearly visible. Include numbers along axes only for bottom left plot of group (a 'group' is an analysis of identical markers).

☒ All plots are contour plots with outliers or pseudocolor plots.

☒ A numerical value for number of cells or percentage (with statistics) is provided.

Methodology

Sample preparation	Isolated brain tumour tissue was digested in cysteine-activated papain (Worthington, 50 U ml-1 in HBSS, pH 7) with 300 U ml-1 DNAse for 5 minutes at 37°C, triturated, and strained through a 70 µm strainer. Cell suspension was resuspended in HBSS and subsequently underlayed with 35% and 70% Percoll (GE Healthcare) and centrifuged for 15 min at 800×g. Interphase between layers was collected and washed using PBS with 1% bovine serum albumin and 0.1% NaN3. After Fc receptor blocking (TruStain FcX™ (Clone 93) #101320, BioLegend), cells were incubated with fluorochrome-conjugated primary antibodies at 1:100 dilution
Instrument	CytoFLEX, Beckman Coulter
Software	CytExpert software for collection of data, FlowJo for analysis
Cell population abundance	Target cell population was relatively small, with TME cells representing only up to 5% of total cell population. After gating out immune cells we split them into smaller subgroups of specific immune cells. To ensure we get enough statistics, we analyzed up to 1-2 million cells for each sample.
Gating strategy	General cell population was selected on FSC/SSC gate, then single cells were selected on FSC-A/FSC-H plot. Single cells population was plotted on CD45-APC and CD11b-PerCP-Cy5.5 and double positive Myeloid cells population was selected. Lymphoid cells were determined as CD45-APC positive, CD11b-PerCP-Cy5.5 negative. Myeloid cells population was gated on Ly6G-V450 and Ly6C-PE-Cy7 plot and separated into three groups: Ly6G high were neutrophils, Ly6C high were monocytes. Ly6G negative, Ly6C low cells were microglia and macrophage mixture. This mixture was separated by CD45-APC expression, where microglial cells express lower level of this surface marker.

☒ Tick this box to confirm that a figure exemplifying the gating strategy is provided in the Supplementary Information.
